# Labour market patterns among women and men following the uptake of their first parental leave benefit in Sweden

**DOI:** 10.1038/s41598-026-35960-1

**Published:** 2026-01-17

**Authors:** Marianna Virtanen, Katalin Gémes, Kristin Farrants, Jakob Bergström, Niklas Gustafsson, Laura Peutere, Ellenor Mittendorfer-Rutz, Kristina Alexanderson

**Affiliations:** 1 Division of Insurance Medicine, Department of Clinical Neuroscience, Karolinska Institutet, 17177 Stockholm, Sweden; 2 School of Educational Sciences and Psychology, University of Eastern Finland, Joensuu, Finland; 3 Social Insurance Institution of Finland (Kela), Helsinki, Finland

**Keywords:** Parental leave, Sick leave, Life course, Prospective, Sequence analysis, Socioeconomic, Diseases, Health care, Medical research, Risk factors

## Abstract

This study identified long-term labour market patterns after taking the first parental leave benefit among women and men in Sweden and the socio-demographic, economic, and health-related characteristics among the identified patterns. We conducted a prospective cohort study, based on nationwide register microdata, including all women (*N* = 43,959) and men (*N* = 43,514) who had their first parental leave benefit uptake in 2010. Sequence analysis was used to explore their labour market patterns over 9 years after parental leave. We identified six labour market clusters for women: ‘*Quick return to employment/studies’* (32%), ‘*Ongoing employment/studies*’ (24%), ‘*Slow return to employment/studies’* (21%), ‘*Weak labour market attachment’* (11%), ‘*Increasing sickness absence/disability pension’* (9%) and ‘*Death/emigration/retirement’* (2%). Among men, there were five clusters: ‘*Ongoing employment/studies*’ (74%), ‘*Weak labour market attachment*’ (13%), ‘*Parental leave*’ (7%), ‘*Increasing sickness absence/disability pension*’ (4%), and ‘*Death/emigration/retirement*’ (2%). Although most were economically active at the end of follow-up, among both women and men, marginalized labour market patterns were characterized by socioeconomic disadvantage and prior morbidity.

**Supplementary Information:**

The online version contains supplementary material available at 10.1038/s41598-026-35960-1.

The transition to parenthood is one of the most significant life events in adulthood, as many structures of the previous life change and there is a new task, raising a child. In high-income countries, work careers among women and men are quite similar before childbirth, but afterwards, there seems to be a differentiation of career paths in such a way that women stay home longer, and their career paths become more fragmented than men’s^1^. To support women’s participation and gender equality in the labour market, the Swedish family policy has emphasized the dual-earner family model with publicly funded childcare^2^, which has been associated with good work-life balance in international comparisons^3^. Another important policy to enable people to combine paid work and family formation is through generous and long parental leave benefits after the birth or adoption of a child^4,5^.

While the purpose of parental leave benefits is to replace parents’ income so they can stay home and care for their child while remaining attached to the labour market and socially insured, taking longer leaves may affect negatively both mothers’ and fathers’ wages^1,6–9^. This can be one of the reasons why, for example in Sweden, fathers still use only approximately 30% of all possible parental leave benefit days^10^. In addition to wages, studies on gender and working life patterns after childbirth have shown disadvantage for women compared to men through part-time work, career interruptions and lower promotion rates^1,6,7,11–13^. Some studies have focused on factors that underlie the likelihood of women and men to take parental leave benefits and the duration of those benefits, finding socioeconomic position and immigrant background to play a role although not in a consistent way^14–18^.

As most previous studies have focused on one outcome at the time, sequence analysis would be informative, as it provides an opportunity to parallelly include several aspects of the life course into long-term sequence trajectories and to consider several states and transitions between them, such as transitions from parental leave to unemployment or from employment to sickness absence (SA) or disability pension (DP)^19,20^. With sequence analysis, it is possible to provide a more holistic view of labour market patterns by incorporating the order, timing, and duration of the main labour market statuses into the main analysis. It also enables extracting hidden patterns that might otherwise go unnoticed. Most of the few previous studies that have used sequence analysis to study labour market outcomes have focused on overall labour market patterns or the work-family life courses among women and men. In the UK, women and men were diverse in older cohorts but became increasingly similar among younger cohorts, because more women engaged in continuous full-time employment^21^. A European-wide study among older individuals suggested that women who had the most traditional life course pattern with early motherhood, multiple children, and non-employment had lower earnings in later life^22^. In the same cohort, women were more likely to shift to part-time or domestic work after childbirth while men maintained full-time employment^23^. Some studies have specifically focused on the labour market patterns around childbirth and found that in Switzerland and Canada, the women’s work careers followed a traditional, male-earner model of family formation^24^, and that among US women, only 36% were identified into a cluster with full-time employment^25^. One study compared the risk of poverty after childbirth in Germany and found that for men, the transition to parenthood had little impact on their economic stability over time whereas for women, the income patterns were heterogeneous, with approximately 59% returning to work quickly and the remaining 41% being economically vulnerable for an extended period of time^26^.

Thus, even though the differential labour market outcomes between women and men after childbirth are well documented in the previous literature, many studies are limited to single outcomes, such as employment or income, and a larger picture including various possible statuses during individual’s life course, such as being absent from work due to illness or being on social security benefits, is lacking. Furthermore, most studies have specifically focused on childbirth or parenthood in general, rather than on the use of parental leave benefits; parenthood does not necessarily imply an interruption in working life, whereas parental leave means taking time off work to take care of a child. Studying labour market patterns after parental leave provides new knowledge about future labour market development after such an interruption.

In the present study, we aimed at capturing the long, multifaceted process of women’s and men’s working lives after their first parental leave in Sweden, using sequence analysis. The overarching aim of this study was to investigate future labour market patterns among women and men who initiated a parental leave benefit, to get a holistic picture of their working life by including sickness absence, disability pension, and being on social security benefits into the models, and to characterize baseline socio-demographic, economic, and health-related factors among the identified labour market patterns. The specific aims of our study were: (1) to identify future nine-year labour market patterns among all women and men in Sweden who had their first recorded parental leave benefit period in 2010; (2) to provide the prevalence of descriptive baseline characteristics for each labour market pattern.

## Methods

### Study population and design

This was a prospective, population- and register-based cohort study of the women and men in Sweden who in 2010 took parental leave benefits for the first time. We used micro-data, linked at individual level from five Swedish nationwide registers. The study cohort included all individuals who were 16–64 years old and lived in Sweden on 31 December 2009, fulfilling the following three inclusion criteria: (1) had lived in Sweden for at least the preceding five years (for those aged 21–64), four years (for those aged 20), or three years (for those aged 16–19); (2) was alive and lived in Sweden through 31 December 2011; and (3) had their first recorded parental leave benefit period during 2010; that is, they had no registered parental leave benefits in Sweden in any year before 2010. The analytical cohort included 43,959 women and 43,514 men, followed up through 2018 (9 years). The project was approved by the Regional Ethical Review Board of Stockholm (2016/1533-32) and by the Swedish Ethical Review Authority (2024-08708-02). As this study was based on register data, there was no obligation for informed consent according to the Swedish legislation and the European Union General Data Protection Regulation (GDPR). All register data were linked and pseudonymised by Statistics Sweden; researchers only had access to de-identified data.

In Sweden, people are entitled to take time off from their work to care for young children, without losing their job. Moreover, parents can together use up to 480 days (approximately 16 months) of parental leave benefit per child (+ 180 days/child with multiple births)^27^. It is possible to use parental leave benefits until the child has finished the first year at compulsory school or when the child turns 8 years, whichever comes last. Parental leave can be for full- or part-time. There were two minor reforms in the parental leave policy during the study years (in 2012 and 2016)^28^.

All residents of Sweden with income from work, unemployment benefits, or parental leave benefits are eligible for sickness absence (SA) benefits if their work capacity is reduced due to morbidity. All residents of Sweden aged 19–64 can be granted disability pension (DP) if their morbidity has led to long-term or permanent reduction of work capacity. Both SA and DP benefits are 80% of lost income, up to a certain level. Both SA and DP can be granted for full- or part-time of ordinary work hours.

### Register data

 We used the following five nationwide administrative registers: (1) The Longitudinal Integrated Database for Labour Market Studies (LISA), kept by Statistics Sweden (data since 1990): age, sex, family situation (married/cohabiting vs. not), birth country (Sweden/not Sweden; the latter including those < 0.01% with missing information), educational level (elementary school [≤ 9 years; including those 0.02% with missing information], high school [10–12 years], university [> 12 years]), type of living area (big city, medium-sized city, small town/municipality), emigration, annual income from paid work or student benefits, and annual number of days with parental leave benefits and unemployment benefits; (2) The National Patient Register (NPR) of inpatient and specialized outpatient healthcare records^29^: dates and main diagnoses of such healthcare, categorizing them into either a somatic or a mental diagnosis (S1 Table); (3) The Prescribed Drug Register^30^: dispensed prescribed medication regarding antidepressant use with Anatomical-Therapeutic-Chemical [ATC] classification codes N05A, N05B, N05C, N06A, N06B, N06C, N07B, and diabetes medication with ATC code A10. From these two databases, the variables indicating a history of diagnosed morbidity (somatic [yes/no], mental [yes/no] for years 2007–2009 were derived (S1 Table); (4) Date of death was obtained from the Cause of Death Register^31^. These three registers are kept by the National Board of Health and Welfare; (5) The Micro-Data for Analysis of the Social Insurance System (MiDAS of the Social Insurance Agency)^32^: Sickness absence and disability pension spells (start and end dates and extent (full- or part-time). We calculated net days for the annual SA and DP days outcome measurement, combining SA and DP; for example, two gross days of 50% benefits were combined to one SA/DP net day.

### Annual main activity state (2010–2018)

We assigned five mutually exclusive labour market states to everyone for each year, based on the economic activity the individual had during most of the year. The state was determined as follows: (1) ‘employed/student’: if the individual had any income from work (as employed or self-employed) or student benefit; (2) ‘on parental leave’: if the sum of net days with parental leave was ≥ 183 days of the year; (3) ‘on SA/DP’, if the sum of the net days with SA/DP benefits was ≥ 183 days of the year; (4) ‘not in the labour market’: (a) if the sum of the net days with unemployment benefits was ≥ 183 days of the year, or (b) if more than 50% of the individual’s annual income came from income support; 5) ‘retired/emigrated/dead’, if the individual had retired with old-age pension (aged 61 or older, 50% or more of the income from old-age pension, and no income from work), or if the individual emigrated or died during the previous year. Thus, a person may have first been identified as belonging to state 1 but then, based on the benefits data, the state could have been re-determined as being 2, 3, or 4. In the case of retirement, emigration or death during the given year, the state was re-determined as being 5.

### Statistical analysis

 We conducted sequence analysis separately for women and men, to identify temporal sequences and to group them into empirically distinct sequence clusters^19,33,34^. The relative frequency of the annual activity states for each year was displayed in state distribution plots. We calculated turbulence and entropy measures for the sequences. To calculate dissimilarity measures between the individual sequences, we used the optimal matching of spell sequences algorithm (“OMspells”) with an expansion cost of 0.5 which yields a sensitivity to both sequencing and duration of spells. To cluster similar sequences, we applied the non-hierarchical cluster algorithm k-medoids on our calculated dissimilarity matrix. Finally, to find the best fitting cluster solution we evaluated the resulting 3–9 cluster solutions by using several partition quality measures with the most emphasis on the weighted average silhouette width (ASWw) and the Hubnert’s C index (C)^35^. We conducted descriptive statistics for the baseline characteristics to present frequencies and proportions of women and men in general and in the different clusters.

The research was carried out according to the declaration of Helsinki and the Strengthening the Reporting of Observational Studies in Epidemiology (STROBE) statement. The sequence analyses were performed using the statistical software R (version 4.4.2) with the package TraMineR (version 2.2.11).

## Results

When we analysed transition probabilities, among both women and men, the highest probablity was not a transition but staying in employment/studies (84.6 and 96.8), followed by a transition from parental leave to employment/studies (71.4 and 81.3), when death/emigration/old-age retirement was not considered (S2 and S3 Tables). This was confirmed by the highest frequencies in those cells. Regarding the longest time spent in each main acitivity state, women spent on average 6.79 years in employment or studies and 1.18 years on parental leave while men spent 8.19 years in employment or studies and 0.53 years not in the labour market state (S4 Table).

The sequence analysis yielded the best fit for a six-cluster solution among women and a five-cluster solution among men (S5 Table). These solutions were further supported by Weighted Average Silhouette Widths (S1 Fig and S2 Fig).

**Fig. 1:**
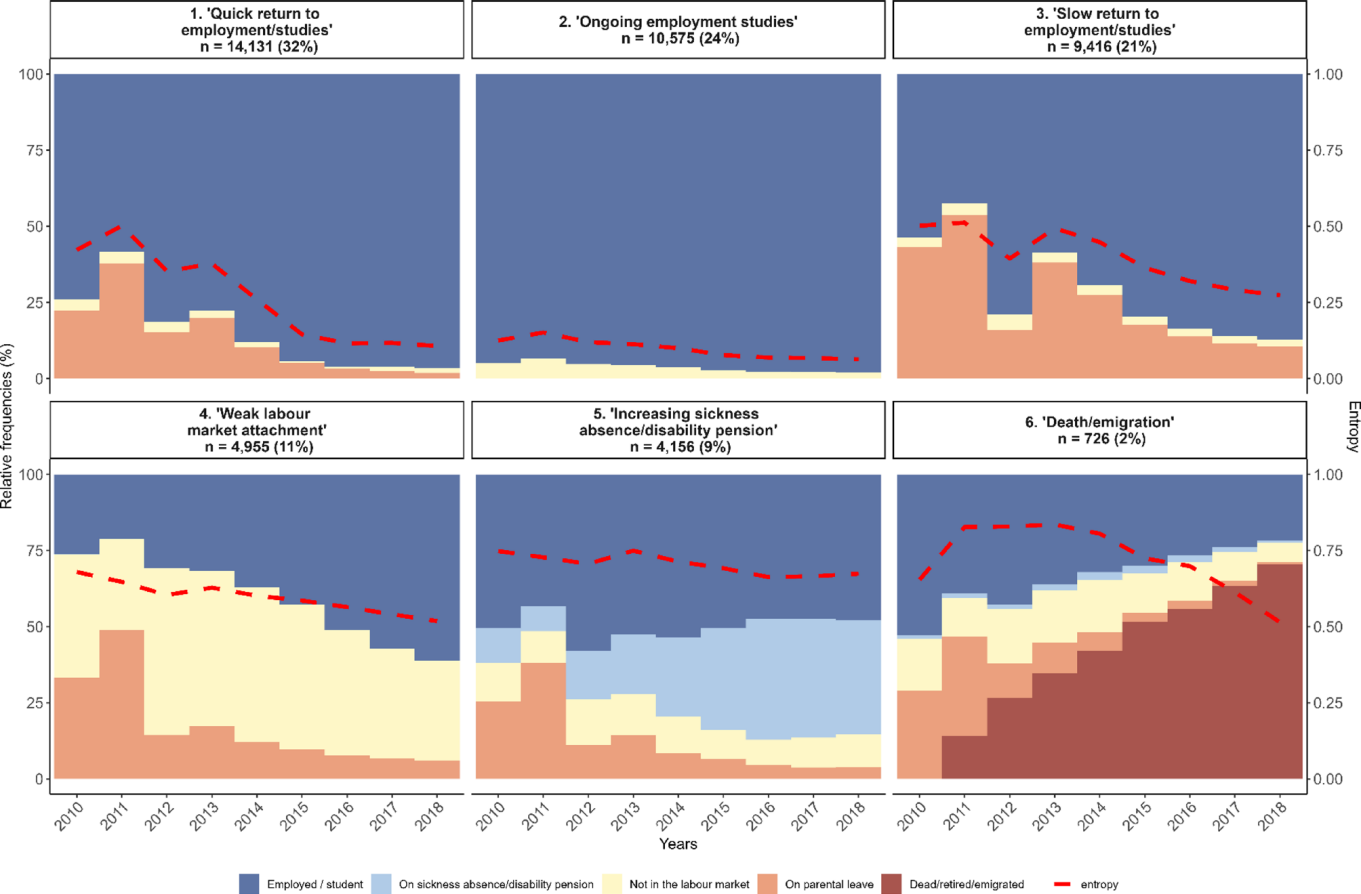
Annual state densities (relative frequencies) in the six-cluster solution among women.

Figure 1 shows the annual state densities within the six identified clusters and their standardized entropy values among women, and S3 Fig shows the corresponding dissected plots. Cluster 1 (hereby named as ‘*Quick return to employment/studies’*) and 2 (named ‘*Ongoing employment/studies*’) were the largest clusters (32% and 24% of all women). Cluster 2 had a low entropy value, which indicates homogeneity of sequences within the cluster. Cluster 3 (named ‘*Slow return to employment/studies’*) and Cluster 4 (named ‘*Weak labour market attachment’*) were smaller (21% and 11% of all women) while the smallest clusters were ‘*Increasing sickness absence/disability pension’* (9%) and ‘*Death/emigration/retirement’* (2%). Clusters 1 and 2 were characterized by high employment rates, although in Custer 1, women had high prevalence of parental leave periods during the 4–5 first years of follow-up. Cluster 3 typically included women who had parental leave throughout the follow-up although at the end, about 90% of them were employed. A high proportion of women in Cluster 4 were out of the labour market in addition to their parental leave periods and in the end, approximately 60% were employed. Cluster 5 was characterized by an increasing proportion of women on sickness absence or disability pension. Of women in Cluster 6, about 75% died, emigrated, or retired by the end of follow-up.

**Fig. 2:**
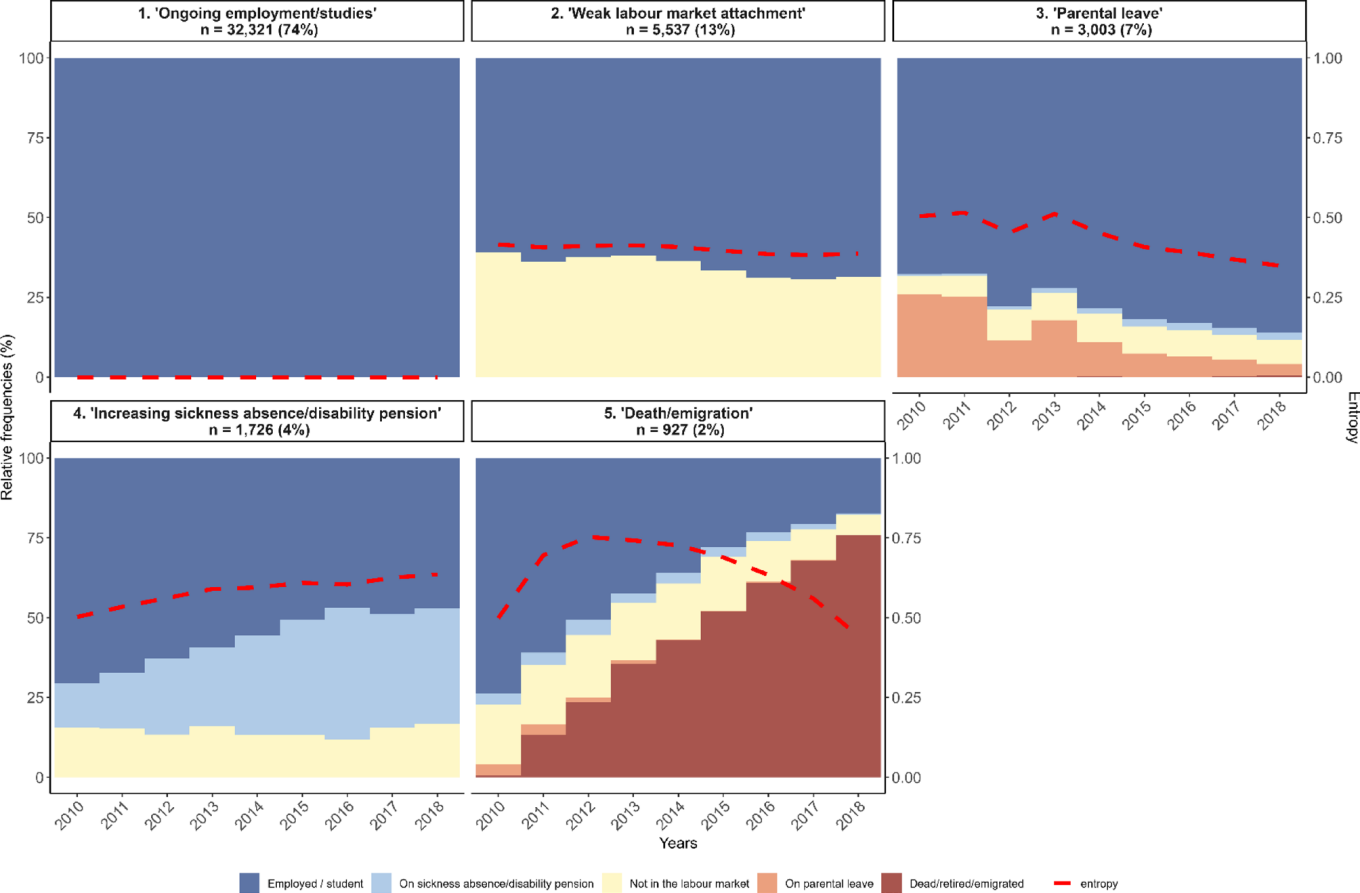
Annual state densities (relative frequencies) in the five-cluster solution among men.

The five-cluster solution among men and their standardized entropy values are shown in Fig. 2, and the corresponding dissected plots in S4 Fig. The largest cluster was Cluster 1 (‘*Ongoing employment/studies*’; 74% of men). This cluster had a very low entropy value, which indicates homogeneity of sequences within the cluster. In Cluster 2 (named ‘*Weak labour market attachment*’; 13% of men), there was a relatively stable (about 30%) proportion of men outside the labour market throughout the follow-up. In Cluster 3 (named ‘*Parental leave’*, 7% of men), about 25% had a parental leave status during 2010 and 2011, with a declining trend replaced by employment or studies thereafter. In Cluster 4 (named ‘*Increasing sickness absence/disability pension*’; 4% of all men), about 20% were outside the labour market and at the end, additional 30% were on sickness absence or disability pension. The smallest cluster was ‘*Death/emigration/retirement*’ (2%); about 75% of men in this cluster either died, emigrated, or retired by the end of follow-up. Sequence turbulence measures for women (S5 Fig.) and men (S6 Fig.) show that women had a higher turbulence value than men, which indicates a more complex and less predictable sequences, while a lower turbulence value for men suggests a simpler and more predictable sequences.

S6 Table (women) and S7 Table (men) show descriptive characteristics for all individuals and by cluster membership. Of the women, 63% were aged 25–34 years, 53% had at least some university studies, 11% were immigrants, 19% had a history of mental morbidity and 51% had a history of somatic morbidity. Correspondingly, 60% of the men were 25–34 years old, 42% had at least some university studies, 13% were immigrants, 11% had a history of mental morbidity, and 37% had a history of somatic morbidity. Among women, Cluster 1 (‘*Quick return to employment/studies’*) was characterized by proportionally more women being married/cohabitant and having higher education and income, higher average duration of parental leave, and less unemployment days and mental and somatic morbidity (S6 Table). In Cluster 2 (‘*Ongoing employment/studies’)* there were more women who were older, single, highly educated, Swedish-born, had shorter parental leave duration, high income, less unemployment days and less mental and somatic morbidity. Cluster 3 *(‘Slow return to employment/studies’*) was characterized by younger age, being married/cohabitant, living in smaller towns or municipalities, having lower education, higher number of parental leave days, lower income, and a history of unemployment. In Cluster 4 (‘*Weak labour market attachment’*) there were more women who were very young (16–24 years), those who were married/cohabitant, and those who lived in smaller municipalities, had lower education, immigrant background, longer duration of parental leave, had no or very low income, and had a history of SA/DP, unemployment or mental morbidity. In Cluster 5 (‘*Increasing sickness absence/disability pension’)*, there were proportionally more women who were older, lived in medium-sized towns or smaller municipalities, had lower education and income, more parental leave days, and a history of unemployment, SA/DP, and mental and somatic morbidity. Cluster 6 (‘*Death/emigration/retirement’)* was characterized by older age, being either not married or not cohabitant, living in big cities, having higher education, immigrant background, slightly shorter duration of parental leave, both high and low income, and a history of SA/DP.

The corresponding descriptive statistics among men are presented in S7 Table. In Cluster 1 *(’Ongoing employment/ studies’)*, there were more men who had higher education, were Swedish-born, had shorter duration of parental leave (although a higher prevalence was found in the class 66–167 days), higher income and less unemployment, SA/DP, or mental or somatic morbidity. Cluster 2 (‘*Weak labour market attachment’*) was characterized by both a younger or older age, lower education, immigrant background, short duration of parental leave, low income, and a history of unemployment, SA/DP, and mental and somatic morbidity. In Cluster 3 (‘*Parental leave’*) there were more men who were less than 35 years old, were either not married or not cohabitant, lived in big cities, had university-level education, and more parental leave days. In Cluster 4 (*‘Increasing sickness absence/disability pension*’), there were proportionally more older men, those either not married or not cohabitant, those with low education, immigrant background, low number of parental leave days, low income, and a history of unemployment, SA/DP, and mental and somatic morbidity. Cluster 5 (‘*Death/emigration/retirement’)* was characterized by older age, being married/cohabitant, living in big cities, both low and high education and income, immigration background, few parental leave days, a history of unemployment, SA/DP, and mental and somatic morbidity.

## Discussion

This register-based prospective cohort study with a nine-year follow-up showed some differences between women and men in their prospective labour market patterns following parental leave in Sweden. Among women, the ongoing employment or studies pattern, i.e., very quick return to work, was found for 24%, whereas 73% had a pattern with a high likelihood of being on parental leave benefit states in several subsequent years. However, at the end of follow-up, approximately 75% of all women were economically active while 23% were not, due to health-related or other reasons. Of the men, 74% had an ongoing work career with few interruptions although we found a small group of men (7%) who spent longer periods on parental leave benefits; at the end of follow-up, most of them returned to work or studies. Of men, fewer (approximately 10%) were not economically active at the end of the follow-up due to health-related or other reasons. Our findings are in line with two previous studies that have used sequence analysis and have shown that women’s working life after childbirth is more fragmented and include less full-time employment than men’s^23–25^.

The socio-demographic, economic, and health-related characteristics by cluster membership suggested that a strong labour market attachment among women (Clusters 1 and 2) was characterized by older age, higher education and income, Swedish-born background, and having less sickness absence/disability pension or physical and mental morbidity. The opposite was evident for clusters leading to a more marginalized labour market position (Clusters 4 and 5), with the exception that women in Cluster 4 (‘*Weak labour market attachment’)* were younger and women in Cluster 5 (‘*Increasing sickness absence/disability pension’)* were typically in the older age groups. In Cluster 3 (‘*Slow return to employment/studies’)*, the women were younger, lived in smaller towns or municipalities, had lower education, higher number of parental leave days, lower income, and a greater likelihood of having history of unemployment but not history of morbidity. Family situation varied, as 65.7% of women in ‘*Ongoing employment and studies*’ cluster were married/cohabitant while the corresponding proportions among women in ‘*Slow return to employment/ studies*’ and ‘*Weak labour market attachment*’ were 77.2% and 77.0%, respectively.

The two labour market patterns ‘*Ongoing employment/studies’* and ‘*Quick return to employment/studies’* together comprised 56% of women. This figure is higher than that reported from one US study (36%)^25^ and approximately similar to another US study (57%)^11^, although due to methodological differences, the studies are not fully comparable. In addition, about 90% among the group ‘*Slow return to employment/studies*’ were active in the labour market nine years later. We did not assess the economic or career-related ‘penalty’ aspect associated with being at home due to childcare, which has been recognized in previous research^6–9,11,13^, but our findings suggest that in Sweden, longer or frequent parental leaves do not necessarily lead to permanent exclusion from the labour market.

In a previous sequence analysis study among the general population of Sweden, 76% of men and 54% of women did not have any interruptions in their work career during a 15-year follow-up^20^. A labour market pattern characterized by interruptions due to parental leave was found for 20% of the participants, mostly women^20^. The present study adds to this research by focusing on the labour market patterns of women and men who initially had an interruption in their work career due to their first recorded parental leave. Our findings suggest that, compared to the national statistics, both women and men with children had a relatively high employment rate after nine years^36^.

In Cluster 4 (‘*Weak labour market attachment’*), there were proportionally more women who lived in smaller municipalities, had low education, immigrant background, long duration of parental leave, had no or very low income, and had a history of SA/DP, unemployment, and mental morbidity. Thus, among women, the baseline socioeconomic and health-related risk factors appeared to be associated with labour market patterns most detached from the labour market, which is in line with a previous study showing that low socioeconomic position and physical and mental morbidity predicted early exit from work due to disability^37^. In our study, immigrant background was associated with a weaker labour market attachment at follow-up, but another study from Sweden showed that immigrant background among mothers was associated with a longer duration of parental leave during the first year after childbirth but not after that^16^.

Among men, the dominant labour market pattern was Cluster 1 (‘*Ongoing employment/studies*’), to which our sequence analysis identified 74% of all men. This pattern was characterized by higher education and income, being Swedish-born, having 66–167 days of parental leave, and having no history of unemployment or health-related issues. Of the three other patterns, two (17% of the men) did not include parental leave as a visible labour market status but were characterized by a weak labour market attachment due to health-related or other reasons. There was also a small Cluster 3 (7%) called ‘*Parental leave’*, in which parental leave status was prevalent during the first follow-up years, eventually ending up to employment or studies. In this cluster, more of the men were younger, had higher education, and lived in big cities. The socio-demographic and economic characteristics found for both Cluster 1 and Cluster 3 are in line with previous study which showed that highly-educated Swedish-born men living in metropolitan areas were more likely to take at least 2 months of parental leave^17^.

Our findings about men being assigned to a continuous labour market pattern and women having more fragmented careers during these nine years are in line with previous findings from Sweden, showing that fathers use a smaller part of all parental leave benefit days^14^. There might be structural characteristics at the workplaces and societal gender norms that place both women and men in more traditional roles even though the social security system encourages gender equality in family life^38^. The fathers’ tendency towards work has been thought to be related to several reasons, such as biological (women breastfeeding), optimizing economic resources in the family, and gender and parenting norms^7,38–41^. Many workplace cultures may still be based on an assumption of an ideal male worker who prioritizes work; this sets limits to fathers’ possibilities to choose to take parental leave^41^. The findings of our study can also be seen from three perspectives: (1) the institutional level of the state, which sets the framework in a form of family policy; (2) the interactional level at the workplace, where negotiations between parenthood and work take place; and (3) the individual level, which refers to an individual decision-making according to institutional level and interactional level circumstances combined with the social context of norms regarding fatherhood and masculinity as well as motherhood and femininity. An interview study focusing on workplace cultures in private sector workplaces in Sweden found that many workplace cultures were still based on an assumption of an ideal male worker who prioritizes work; this sets limits to fathers’ possibilities to choose to take parental leave^41^. Thus, the workplace culture is an important topic for future studies.

Strengths of this study include the longitudinal cohort study design based on the entire population of Sweden, the use of data from high-quality registers with no loss to follow-up, the administrative data which is not hampered by recall bias^29,32,42^. Limitations in sequence analysis relate to researcher judgement when choosing dissimilarity functions and clustering methods. However, alternative methods to sequence analysis, such as survival or group-based trajectory analysis do not capture the richness of individuals’ life courses as an entity^43^. No conclusions about the direction of causality can be made based on this observational study, as the sequence analysis method is highly explorative and descriptive in nature. Our definition of states was also a simplified representation of people’s labour market sequences, limited by data availability and because it was important to keep the number of states reasonable. We defined the main activity on an annual basis, as some of the variables were available on an annual basis only. As especially women’s labour market patterns after childbirth can be fragmented, further research with more detailed register data (e.g., monthly) is warranted. Identification of the first parental leave benefit uptake was based on register data. About 25% of fathers in Sweden did not take parental leave benefits they were eligible for in 2010^18^, so their labour market patterns were not possible to observe in this study. Studying the uptake of the first parental leave benefit is important, as it indicates a temporary absence from work and research can identify factors that are associated with longer absence, subsequent withdrawal from work and possible differences in labour market patterns among women and men, after having used parental leave benefits.

Information on morbidity was based on treatment in secondary (specialized) healthcare and purchased prescribed medications, thus, much of the morbidity treated in primary healthcare or not treated at all was not accounted for. In addition, as this study was based on specific register data, we could not include other characteristics that are likely to affect labour market patterns, such as insecure jobs, social support network, personality traits, individual preferences, workplace culture, motivation related to working career and family situation, including the number of children. For example, previous research has shown that family-related events, such as divorce, were associated with a higher risk of labour market marginalization^44^ and that lack of employment and having a temporary contract were associated with slower and weaker labour market attachment after birth of the first child^12^. These are important topics for future studies, as they could provide ideas for interventions and practical implications. In future studies, it is also important to examine more fine-grained labour market states that account for, e.g., job quality, which is an important predictor of early exit from the labour market^45^. This study did not specifically examine the impact of policy reforms on parental leave benefit uptake among different socioeconomic and age groups, which could be a topic of future studies. Finally, our findings are generalizable to Sweden in this specific period.

In conclusion, this study carried out in a Swedish context shows that 74% of men had few interruptions in their work careers after taking their first parental leave benefit whereas a similar pattern was found for only 24% of women. However, even though women spent more time on parental leave, nine years later, most of them participated in the labour market equally with men. Among both women and men, there were sub-groups with a more disadvantaged labour market development which was characterized by socioeconomic disadvantage and prior morbidity.

## Supplementary Information

Below is the link to the electronic supplementary material.

Supplementary Material 1

## Data Availability

The used data cannot be made publicly available due to privacy regulations. According to the General Data Protection Regulation, the Swedish law SFS 2018:218, the Swedish Data Protection Act, the Swedish Ethical Review Act, and the Public Access to Information and Secrecy Act, these types of sensitive data can only be made available for specific purposes that meets the criteria for access to this type of sensitive and confidential data as determined by a legal review. Contact email for data requests: [imas-cns@ki.se](mailto: imas-cns@ki.se) or Prof. Ellenor Mittendorfer-Rutz ( [ellenor.mittendorfer-rutz@ki.se](mailto: ellenor.mittendorfer-rutz@ki.se) ).
