# Supplementary material for: Labour market patterns among women and men following the uptake of their first parental leave benefit in Sweden
Source: Sci Rep. 2026 Jan 17;16:2595. doi: 10.1038/s41598-026-35960-1 (PMC12819409; doi:10.1038/s41598-026-35960-1)

,

**S1 Table.** Data sources and diagnostic groups used to define a history of somatic or mental morbidity (2007-2009)

| Disease | Data source, diagnostic group and ICD-10 codes |
|---|---|
| Somatic disease or injury | National Patient Register (NPR): |
| | Certain infectious and parasitic diseases (A00-B99); |
| | Neoplasms (C00-D48); |
| | Diabetes (E10-E14); |
| | Other endocrine, nutritional and metabolic diseases (E00-E09, E15-E90); |
| | Diseases of the nervous system (G00-G99); |
| | Diseases of the eye and adnexa (H00-H59); |
| | Diseases of the circulatory system (I00-I99); |
| | Diseases of the respiratory system (J00-J99); |
| | Diseases of the digestive system (K00-K93); |
| | Diseases of the skin and subcutaneous tissue (L00-L99); |
| | Musculoskeletal disorders (M00-M99); |
| | Injuries (S00-T35, T66-T78, T79); |
| | Poisonings (T36-T65); |
| | Pregnancy, childbirth and the puerperium; (O00-O02, O08-O75, O82, O84.2, O85-O91, O94-O99) |
| | Other: any of N, P, Q, R, V, Y, U04, U07 |
| | Prescribed Drug Register: |
| | Psychopharmaceutical prescriptions (Anatomical-Therapeutic-Chemical [ATC] classification code A10 for diabetes medication. |
| Mental disorder | National Patient Register (NPR): |
| | Mental disorders (F00-F99, Z73.0); |
| | Prescribed Drug Register: |
| | ATC codes N05A, N05B, N05C, N06A, N06B, N06C, N07B) for psychopharmaceutical medication |

**S2 Table**. Transition probabilities between states and their frequencies among women

| **Probability (%)** | **[-> Death, old-age pension, emigration]** | **[-> Parental leave]** | **[-> Not in the labour market]** | **[-> SA/DP]** | **[-> Employment /studies]** |
|---|---|---|---|---|---|
| [Death, old-age pension, emigration ->] | 88.7 | 0.5 | 5.3 | 0 | 5.5 |
| [Parental leave ->] | 0.3 | 13.5 | 13.4 | 1.4 | 71.4 |
| [Not in the labour market ->] | 0.8 | 15.9 | 47.5 | 2.1 | 33.7 |
| [SA/DP ->] | 0.5 | 8.4 | 8.1 | 58.5 | 24.6 |
| [Employment/studies ->] | 0.1 | 11.3 | 2.8 | 1.2 | 84.6 |

| **Frequency (n)** | **[-> Death, old-age pension, emigration]** | **[-> Parental leave]** | **[-> Not in the labour market]** | **[-> SA/DP]** | **[-> Employment /studies]** |
|---|---|---|---|---|---|
| [Death, old-age pension, emigration ->] | 1,855 | 10 | 110 | <5 | 115 |
| [Parental leave ->] | 139 | 6,756 | 6,736 | 698 | 35,770 |
| [Not in the labour market ->] | 239 | 4,828 | 14,462 | 649 | 10,272 |
| [SA/DP ->] | 37 | 684 | 662 | 4,766 | 2,004 |
| [Employment/studies ->] | 332 | 29,415 | 7,221 | 3,110 | 220,801 |

*Note*. SA/DP, sickness absence/disability pension.

,

**S3 Table**. Transition probabilities between states and their frequencies among men

| **Probability (%)** | **[-> Death, old-age pension, emigration]** | **[-> Parental leave]** | **[-> Not in the labour market]** | **[-> SA/DP]** | **[-> Employment /studies]** |
|---|---|---|---|---|---|
| [Death, old-age pension, emigration ->] | 90.4 | 0 | 4.1 | 0.1 | 5.3 |
| [Parental leave ->] | 0.7 | 4.5 | 11.7 | 1.7 | 81.3 |
| [Not in the labour market ->] | 1.7 | 1 | 64.5 | 1.9 | 30.9 |
| [SA/DP ->] | 1.5 | 0.7 | 10.8 | 64.6 | 22.4 |
| [Employment/studies ->] | 0.2 | 0.7 | 1.9 | 0.5 | 96.8 |

| **Frequency (n)** | **[-> Death, old-age pension, emigration]** | **[-> Parental leave]** | **[-> Not in the labour market]** | **[-> SA/DP]** | **[-> Employment /studies]** |
|---|---|---|---|---|---|
| [Death, old-age pension, emigration ->] | 2,512 | <5 | 115 | <5 | 148 |
| [Parental leave ->] | 25 | 154 | 399 | 57 | 2,762 |
| [Not in the labour market ->] | 344 | 206 | 13,390 | 401 | 6,419 |
| [SA/DP ->] | 68 | 31 | 483 | 2,890 | 1,003 |
| [Employment/studies ->] | 541 | 2,303 | 5,917 | 1,529 | 306,412 |

*Note*. SA/DP, sickness absence/disability pension.

,

**S4 Table**. Mean time spent in each state as main activity among women and men

| **Women** | **Mean (years)** |
|---|---|
| Death, old-age pension, emigration | 0.06 |
| Parental leave | 1.18 |
| Not in the labour market | 0.76 |
| SA/DP | 0.22 |
| Employment/studies | 6.79 |
| | |
| **Men** | |
| Death, old-age pension, emigration | 0.08 |
| Parental leave | 0.08 |
| Not in the labour market | 0.53 |
| SA/DP | 0.12 |
| Employment/studies | 8.19 |

*Note*. SA/DP, sickness absence/disability pension.

’

**S5 Table.** Quality indices for different number of cluster solutions derived from sequence analysis for women and men

| | PBC | HG | HGSD | ASW | ASWw | CH | R2 | CHsq | R2sq | HC | Clusters |
|---|---|---|---|---|---|---|---|---|---|---|---|
| Women | 0,5 | 0,92 | 0,92 | 0,68 | 0,68 | 12567 | 0,22 | 19486 | 0,31 | 0,24 | 2 |
| | 0,45 | 0,83 | 0,83 | 0,5 | 0,5 | 12524 | 0,36 | 15568 | 0,41 | 0,13 | 3 |
| | 0,55 | 0,88 | 0,88 | 0,54 | 0,54 | 15275 | 0,51 | 60201 | 0,8 | 0,04 | 4 |
| | 0,4 | 0,77 | 0,77 | 0,48 | 0,48 | 16977 | 0,61 | 53417 | 0,83 | 0,06 | 5 |
| | 0,35 | 0,77 | 0,77 | 0,54 | 0,54 | 16324 | 0,65 | 49315 | 0,85 | 0,05 | 6 |
| | 0,33 | 0,84 | 0,84 | 0,59 | 0,59 | 16844 | 0,7 | 45690 | 0,86 | 0,03 | 7 |
| | 0,34 | 0,86 | 0,86 | 0,6 | 0,6 | 16672 | 0,73 | 57428 | 0,9 | 0,03 | 8 |
| | 0,34 | 0,9 | 0,9 | 0,64 | 0,64 | 16512 | 0,75 | 55000 | 0,91 | 0,02 | 9 |
| Men | 0,71 | 0,93 | 0,93 | 0,87 | 0,87 | 29305 | 0,4 | 49989 | 0,53 | 0,08 | 2 |
| | 0,81 | 0,95 | 0,95 | 0,88 | 0,88 | 29229 | 0,57 | 52873 | 0,71 | 0,06 | 3 |
| | 0,65 | 0,95 | 0,95 | 0,82 | 0,82 | 33225 | 0,7 | 43569 | 0,75 | 0,05 | 4 |
| | 0,69 | 0,99 | 0,99 | 0,89 | 0,89 | 43569 | 0,8 | 67020 | 0,86 | 0,01 | 5 |
| | 0,69 | 0,99 | 0,99 | 0,9 | 0,9 | 43381 | 0,83 | 80582 | 0,9 | 0 | 6 |
| | 0,69 | 1 | 1 | 0,93 | 0,93 | 42577 | 0,85 | 71072 | 0,91 | 0 | 7 |
| | 0,69 | 1 | 1 | 0,93 | 0,93 | 43099 | 0,87 | 81839 | 0,93 | 0 | 8 |
| | 0,69 | 1 | 1 | 0,94 | 0,94 | 42420 | 0,89 | 96658 | 0,95 | 0 | 9 |

*Note*. PCB, Point Biserial Correlation; HG, Hubert’s Gamma; HGSD, Hubert’s Somers’ D; ASW, Average Silhouette Width; ASWw, Average Silhouette Width (weighted); CH, Calinski-Harabász index; R2, Pseudo $R^2$; CHsq, Calinski-Harabász index squared; R2sq, Pseudo $R^2$ squared; HC, Hubert’s C.

,

**S6 Table.** Characteristics of women at baseline by cluster membership, n (%)

| Characteristics | Total n=43,959 | Cluster 1 ('Quick return to employment /studies') n=14,131 | Cluster 2 ('Ongoing employment /studies) n=10,575 | Cluster 3 ('Slow return to employment/ studies') n=9416 | Cluster 4 ('Weak labour market attachment') n=4955 | Cluster 5 ('Increasing sickness absence /disability pension') n=4156 | Cluster 6 ('Death/ emigration /retirement') n=726 |
|---|---|---|---|---|---|---|---|
| Age group (years) | | | | | | | |
| 16-24 | 11,073 (25.2) | 2,879 (20.4) | 1,079 (10.2) | 3,223 (34.2) | 2,533 (51.1) | 1,224 (29.5) | 135 (18.6) |
| 25-34 | 27,538 (62.6) | 9,448 (66.9) | 7,553 (71.4) | 5,787 (61.5) | 1,973 (39.8) | 2,301 (55.4) | 476 (65.6) |
| 35-44 | 5,348 (12.2) | 1,804 (12.8) | 1,943 (18.4) | 406 (4.3) | 449 (9.1) | 631 (15.2) | 115 (15.8) |
| Family situation in 2009 | | | | | | | |
| Married/cohabited | 32,078 (73.0) | 10,540 (74.6) | 6,951 (65.7) | 7,268 (77.2) | 3,816 (77.0) | 3,036 (73.1) | 467 (64.3) |
| Not married/cohabited | 11,881 (27.0) | 3,591 (25.4) | 3,624 (34.3) | 2,148 (22.8) | 1,139 (23.0) | 1,120 (26.9) | 259 (35.7) |
| Type of region | | | | | | | |
| Big city | 21,371 (48.6) | 6,907 (48.9) | 5,847 (55.3) | 4,146 (44.0) | 2,158 (43.6) | 1,844 (44.4) | 469 (64.6) |
| Medium sized city | 15,959 (36.3) | 5,108 (36.1) | 3,504 (33.1) | 3,627 (38.5) | 1,884 (38.0) | 1,643 (39.5) | 193 (26.6) |
| Smaller municipality | 6,629 (15.1) | 2,116 (15.0) | 1,224 (11.6) | 1,643 (17.4) | 913 (18.4) | 669 (16.1) | 64 (8.8) |
| Educational level | | | | | | | |
| University | 23,116 (52.6) | 7,919 (56.0) | 7,743 (73.2) | 4,227 (44.9) | 1,151 (23.2) | 1,658 (39.9) | 418 (57.6) |
| High school | 16,647 (37.9) | 5,530 (39.1) | 2,564 (24.2) | 4,387 (46.6) | 2,226 (44.9) | 1,739 (41.8) | 201 (27.7) |
| Elementary (incl. missing information) | 4,196 (9.5) | 682 (4.8) | 268 (2.5) | 802 (8.5) | 1,578 (31.8) | 759 (18.3) | 107 (14.7) |
| Immigration status | | | | | | | |
| Sweden | 39,251 (89.3) | 12,866 (91.0) | 9,746 (92.2) | 8,529 (90.6) | 3,876 (78.2) | 3,696 (88.9) | 538 (74.1) |
| Not Sweden (incl. missing information) | 4,708 (10.7) | 1,265 (9.0) | 829 (7.8) | 887 (9.4) | 1,079 (21.8) | 460 (11.1) | 188 (25.9) |
| Parental leave days (2010-11) | | | | | | | |
| 1-<66 | 656 (1.5) | 107 (0.8) | 300 (2.8) | 16 (0.2) | 74 (1.5) | 121 (2.9) | 38 (5.2) |
| 66-<168 | 4,733 (10.8) | 1,033 (7.3) | 2,707 (25.6) | 117 (1.2) | 310 (6.3) | 457 (11.0) | 109 (15.0) |
| 168-<273 | 17,262 (39.3) | 5,875 (41.6) | 6,167 (58.3) | 2,505 (26.6) | 1,113 (22.5) | 1,369 (32.9) | 233 (32.1) |
| ≥273 | 21,308 (48.5) | 7,116 (50.4) | 1,401 (13.2) | 6,778 (72.0) | 3,458 (69.8) | 2,209 (53.2) | 346 (47.7) |
| Income from employment (2009), SEK | | | | | | | |
| 0 | 2,944 (6.7) | 267 (1.9) | 212 (2.0) | 324 (3.4) | 1,373 (27.7) | 627 (15.1) | 141 (19.4) |
| 0<-<85600 | 5,785 (13.2) | 1,302 (9.2) | 778 (7.4) | 1,224 (13.0) | 1,587 (32.0) | 780 (18.8) | 114 (15.7) |

| | | | | | | | |
|---|---|---|---|---|---|---|---|
| 85600-<171200 | 5,306 (12.1) | 1,561 (11.0) | 869 (8.2) | 1,385 (14.7) | 803 (16.2) | 622 (15.0) | 66 (9.1) |
| 171200-<321000 | 20,731 (47.2) | 7,738 (54.8) | 4,967 (47.0) | 5,114 (54.3) | 1,033 (20.8) | 1,679 (40.4) | 200 (27.5) |
| 321000-<428000 | 6,535 (14.9) | 2,365 (16.7) | 2,575 (24.3) | 1,003 (10.7) | 119 (2.4) | 344 (8.3) | 129 (17.8) |
| ≥428000 | 2,658 (6.0) | 898 (6.4) | 1,174 (11.1) | 366 (3.9) | 40 (0.8) | 104 (2.5) | 76 (10.5) |
| Number of SA/DP days (2009) | | | | | | | |
| 0 | 25,405 (57.8) | 8,262 (58.5) | 5,959 (56.3) | 5,659 (60.1) | 2,596 (52.4) | 2,663 (64.1) | 266 (36.6) |
| 0-<90 | 17,015 (38.7) | 5,626 (39.8) | 4,475 (42.3) | 3,618 (38.4) | 2,163 (43.7) | 705 (17.0) | 428 (59.0) |
| 90-<180 | 657 (1.5) | 182 (1.3) | 97 (0.9) | 96 (1.0) | 77 (1.6) | 196 (4.7) | 9 (1.2) |
| 180-365 | 882 (2.0) | 61 (0.4) | 44 (0.4) | 43 (0.5) | 119 (2.4) | 592 (14.2) | 23 (3.2) |
| Number of unemployment days (2009) | | | | | | | |
| 0 | 36,696 (83.5) | 12,568 (88.9) | 9,673 (91.5) | 7,925 (84.2) | 2,803 (56.6) | 3,122 (75.1) | 605 (83.3) |
| 0-<90 | 2,943 (6.7) | 807 (5.7) | 440 (4.2) | 678 (7.2) | 627 (12.7) | 343 (8.3) | 48 (6.6) |
| 90-<180 | 1,859 (4.2) | 383 (2.7) | 222 (2.1) | 403 (4.3) | 549 (11.1) | 269 (6.5) | 33 (4.5) |
| 180-365 | 2,461 (5.6) | 373 (2.6) | 240 (2.3) | 410 (4.4) | 976 (19.7) | 422 (10.2) | 40 (5.5) |
| History of mental disorder | | | | | | | |
| No | 35,793 (81.4) | 12,025 (85.1) | 9,111 (86.2) | 8,018 (85.2) | 3,715 (75.0) | 2,344 (56.4) | 580 (79.9) |
| Yes | 8,166 (18.6) | 2,106 (14.9) | 1,464 (13.8) | 1,398 (14.8) | 1,240 (25.0) | 1,812 (43.6) | 146 (20.1) |
| History of somatic disease | | | | | | | |
| No | 21,544 (49.0) | 7,273 (51.5) | 5,465 (51.7) | 4,745 (50.4) | 2,315 (46.7) | 1,393 (33.5) | 353 (48.6) |
| Yes | 22,415 (51.0) | 6,858 (48.5) | 5,110 (48.3) | 4,671 (49.6) | 2,640 (53.3) | 2,763 (66.5) | 373 (51.4) |

*Note*. SA/DP, sickness absence /disability pension; SEK, Swedish krona.

**S7 Table.** Characteristics of men at baseline by cluster membership, n (%)

| Characteristics | Total n=43,514 | Cluster 1 ('Ongoing employment/ studies) n=32,321 | Cluster 2 ('Weak labour market attachment') n=5537 | Cluster 3 ('Parental leave') n=3003 | Cluster 4 ('Increasing sickness absence /disability pension') n=1726 | Cluster 5 ('Death/ retirement/ emigration') n=927 |
|---|---|---|---|---|---|---|
| Age group (years) | | | | | | |
| 16-24 | 3,469 (8.0) | 2,242 (6.9) | 713 (12.9) | 291 (9.7) | 175 (10.1) | 48 (5.2) |
| 25-34 | 26,266 (60.4) | 20,357 (63.0) | 2,736 (49.4) | 1,930 (64.3) | 826 (47.9) | 417 (45.0) |
| 35-44 | 11,944 (27.4) | 8,795 (27.2) | 1,654 (29.9) | 696 (23.2) | 533 (30.9) | 266 (28.7) |
| 45-64 | 1,835 (4.2) | 927 (2.9) | 434 (7.8) | 86 (2.9) | 192 (11.1) | 196 (21.1) |
| Family situation in 2009 | | | | | | |
| Married/cohabited | 31,681 (72.8) | 23,775 (73.6) | 3,950 (71.3) | 2,052 (68.3) | 1,185 (68.7) | 719 (77.6) |
| Not married/cohabited | 11,833 (27.2) | 8,546 (26.4) | 1,587 (28.7) | 951 (31.7) | 541 (31.3) | 208 (22.4) |
| Type of region | | | | | | |
| Big city | 19,960 (45.9) | 14,490 (44.8) | 2,533 (45.7) | 1,749 (58.2) | 683 (39.6) | 505 (54.5) |
| Medium sized city | 16,519 (38.0) | 12,509 (38.7) | 2,090 (37.7) | 909 (30.3) | 685 (39.7) | 326 (35.2) |
| Smaller municipality | 7,035 (16.2) | 5,322 (16.5) | 914 (16.5) | 345 (11.5) | 358 (20.7) | 96 (10.4) |
| Educational level | | | | | | |
| University | 18,194 (41.8) | 14,200 (43.9) | 1,590 (28.7) | 1,546 (51.5) | 385 (22.3) | 473 (51.0) |
| High school | 20,903 (48.0) | 15,881 (49.1) | 2,671 (48.2) | 1,144 (38.1) | 918 (53.2) | 289 (31.2) |
| Elementary (incl. missing information) | 4,417 (10.2) | 2,240 (6.9) | 1,276 (23.0) | 313 (10.4) | 423 (24.5) | 165 (17.8) |
| Immigration status | | | | | | |
| Sweden | 37,680 (86.6) | 29,200 (90.3) | 3,964 (71.6) | 2,570 (85.6) | 1,352 (78.3) | 594 (64.1) |
| Not Sweden (incl. missing information) | 5,834 (13.4) | 3,121 (9.7) | 1,573 (28.4) | 433 (14.4) | 374 (21.7) | 333 (35.9) |
| Parental leave days (2010-11) | | | | | | |
| 1-<66 | 20,977 (48.2) | 15,958 (49.4) | 3,154 (57.0) | 378 (12.6) | 994 (57.6) | 493 (53.2) |
| 66-<168 | 17,180 (39.5) | 13,661 (42.3) | 1,862 (33.6) | 748 (24.9) | 601 (34.8) | 308 (33.2) |
| 168-<273 | 4,662 (10.7) | 2,620 (8.1) | 490 (8.8) | 1,341 (44.7) | 124 (7.2) | 87 (9.4) |
| ≥273 | 695 (1.6) | 82 (0.3) | 31 (0.6) | 536 (17.8) | 7 (0.4) | 39 (4.2) |
| Income from employment (2009) SEK | | | | | | |
| 0 | 1,739 (4.0) | 251 (0.8) | 898 (16.2) | 159 (5.3) | 285 (16.5) | 146 (15.7) |
| 0<-<85600 | 2,431 (5.6) | 957 (3.0) | 942 (17.0) | 230 (7.7) | 218 (12.6) | 84 (9.1) |

,

| | | | | | | |
|---|---|---|---|---|---|---|
| 85600-<171200 | 2,997 (6.9) | 1,603 (5.0) | 826 (14.9) | 262 (8.7) | 224 (13.0) | 82 (8.8) |
| 171200-<321000 | 17,466 (40.1) | 13,566 (42.0) | 1,880 (34.0) | 1,163 (38.7) | 642 (37.2) | 215 (23.2) |
| 321000-<428000 | 11,704 (26.9) | 9,985 (30.9) | 544 (9.8) | 747 (24.9) | 258 (14.9) | 170 (18.3) |
| ≥428000 | 7,177 (16.5) | 5,959 (18.4) | 447 (8.1) | 442 (14.7) | 99 (5.7) | 230 (24.8) |
| Number of SA/DP days (2009) | | | | | | |
| 0 | 19,425 (44.6) | 13,813 (42.7) | 2,781 (50.2) | 1,346 (44.8) | 1,164 (67.4) | 321 (34.6) |
| 0<-90 | 23,193 (53.3) | 18,296 (56.6) | 2,583 (46.6) | 1,592 (53.0) | 174 (10.1) | 548 (59.1) |
| 90<-180 | 295 (0.7) | 146 (0.5) | 59 (1.1) | 21 (0.7) | 62 (3.6) | 7 (0.8) |
| 180-365 | 601 (1.4) | 66 (0.2) | 114 (2.1) | 44 (1.5) | 326 (18.9) | 51 (5.5) |
| Number of unemployment days (2009) | | | | | | |
| 0 | 37,323 (85.8) | 29,548 (91.4) | 3,232 (58.4) | 2,536 (84.4) | 1,251 (72.5) | 756 (81.6) |
| 0-<90 | 2,038 (4.7) | 1,226 (3.8) | 500 (9.0) | 143 (4.8) | 119 (6.9) | 50 (5.4) |
| 90-<180 | 1,499 (3.4) | 783 (2.4) | 468 (8.5) | 112 (3.7) | 108 (6.3) | 28 (3.0) |
| 180-365 | 2,654 (6.1) | 764 (2.4) | 1,337 (24.1) | 212 (7.1) | 248 (14.4) | 93 (10.0) |
| History of mental disorder | | | | | | |
| No | 38,854 (89.3) | 29,787 (92.2) | 4,563 (82.4) | 2,657 (88.5) | 1,080 (62.6) | 767 (82.7) |
| Yes | 4,660 (10.7) | 2,534 (7.8) | 974 (17.6) | 346 (11.5) | 646 (37.4) | 160 (17.3) |
| History of somatic disease | | | | | | |
| No | 27,370 (62.9) | 20,944 (64.8) | 3,187 (57.6) | 1,918 (63.9) | 755 (43.7) | 566 (61.1) |
| Yes | 16,144 (37.1) | 11,377 (35.2) | 2,350 (42.4) | 1,085 (36.1) | 971 (56.3) | 361 (38.9) |

*Note*. SA/DP, sickness absence/disability pension; SEK, Swedish krona.

,

**S1 Fig.** Weighted Average Silhouette Width plotted by number of clusters among women

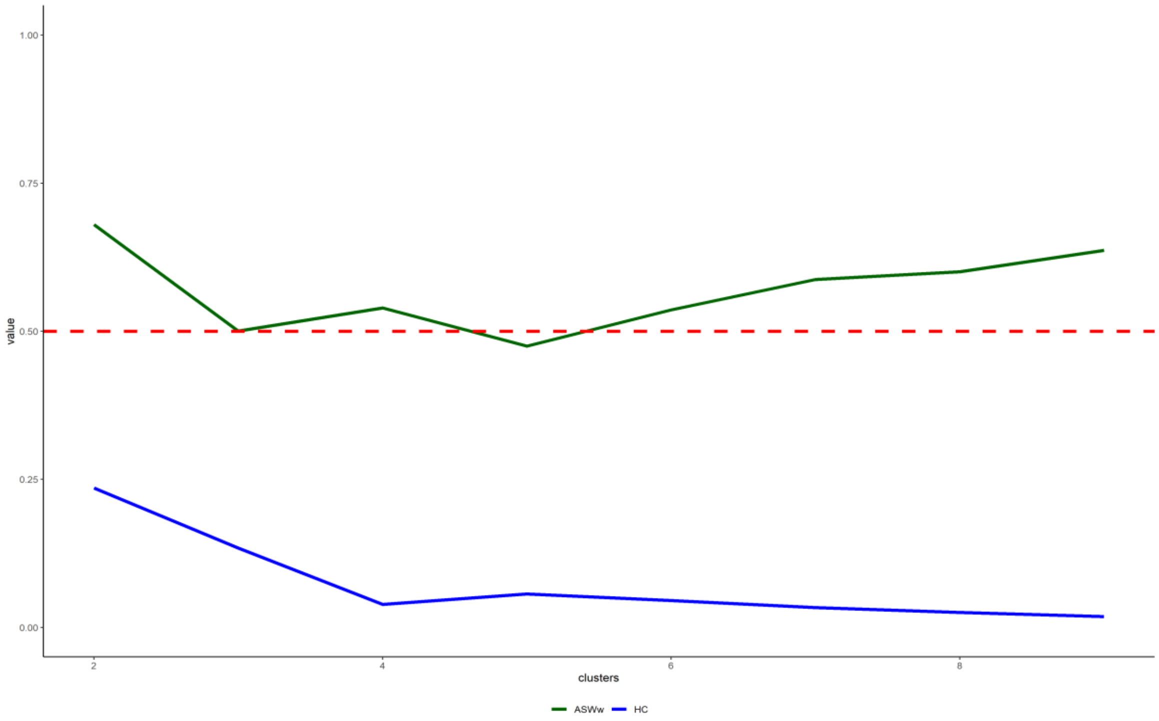

,

**S2 Fig.** Weighted Average Silhouette Width plotted by number of clusters among men

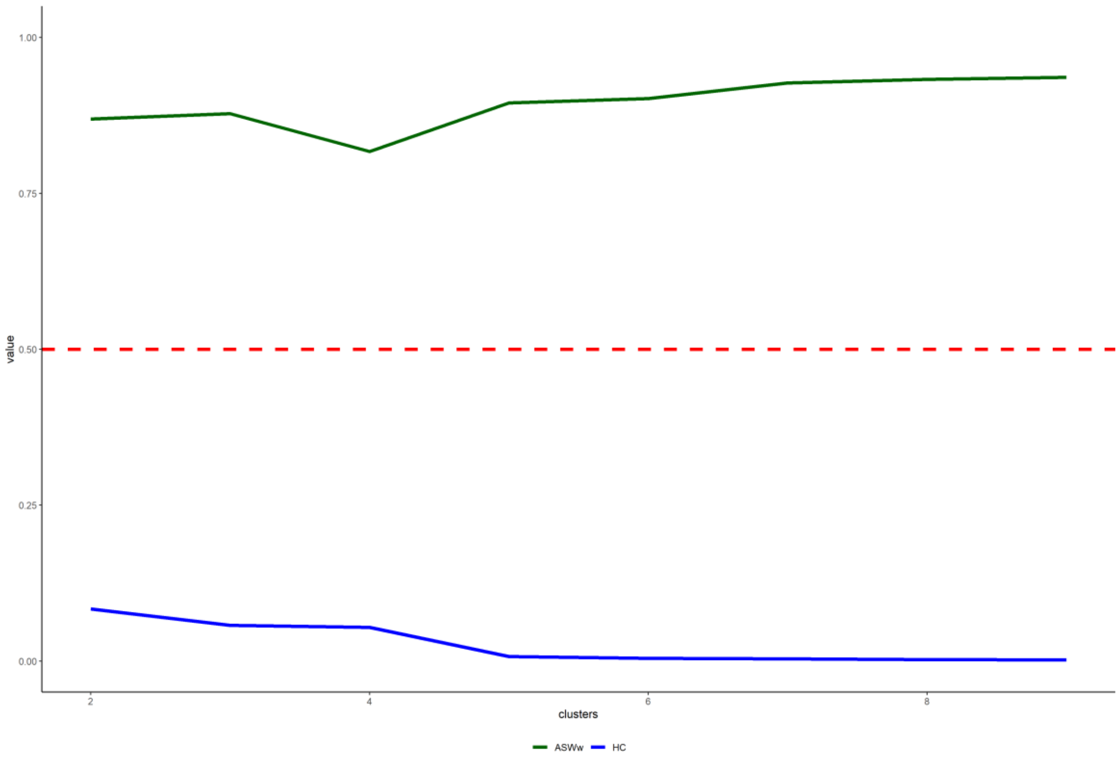

,

**S3 Fig.** Dissected plots; relative frequencies of the different states over study years by clusters among women

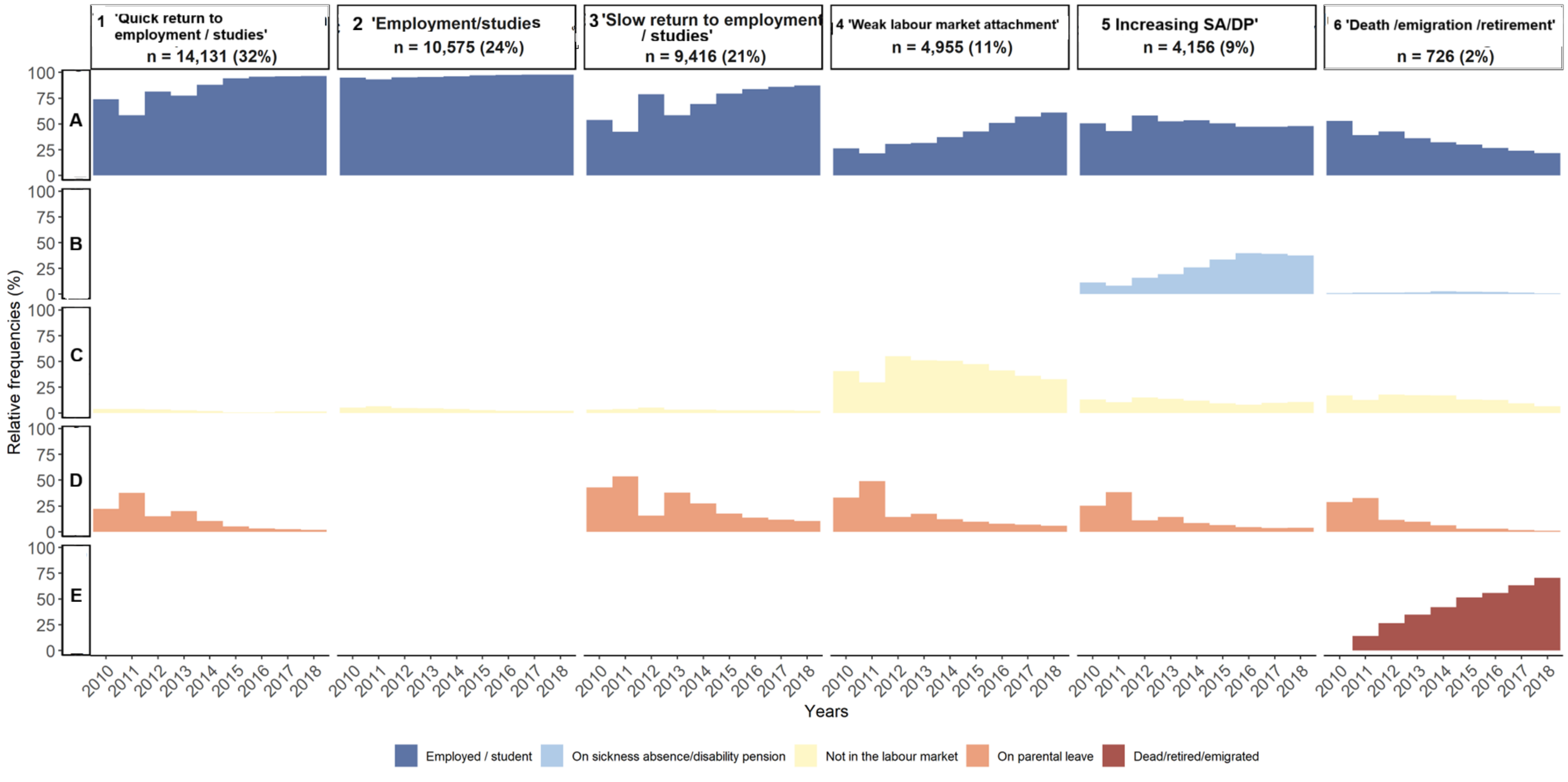


A=Employed/student; B=On sickness absence/disability pension (SA/DP); C=Not in the labour market; D=On parental leave; E=Dead/emigrated/retired

,

**S4 Fig.** Dissected plots – Relative frequencies of the different states over study years by clusters among men

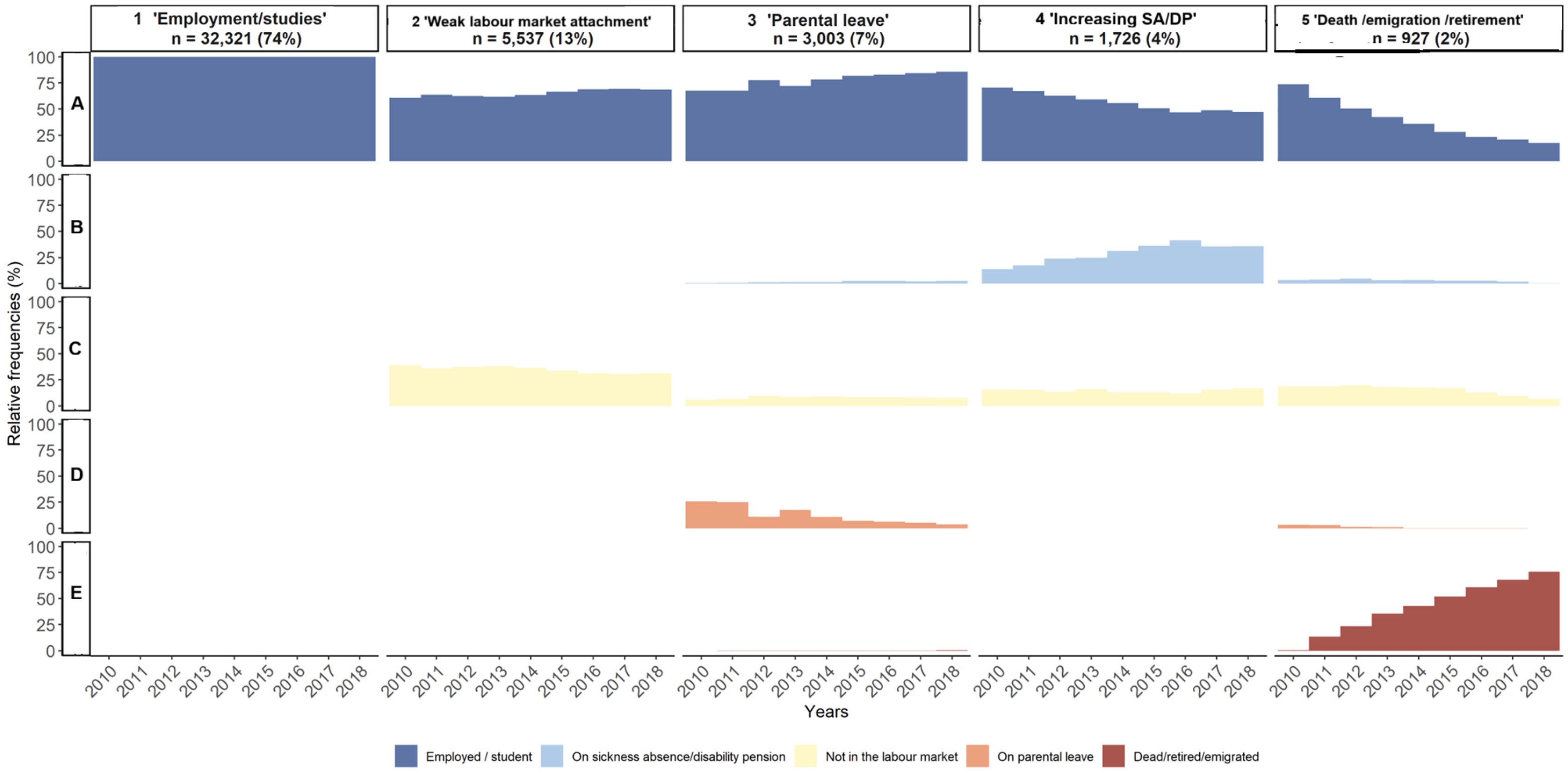


A=Employed/student; B=On sickness absence/disability pension (SA/DP); C=Not in the labour market; D=On parental leave; E=Dead/emigrated/retired

,

**S5 Fig.** Sequence turbulence measures for women

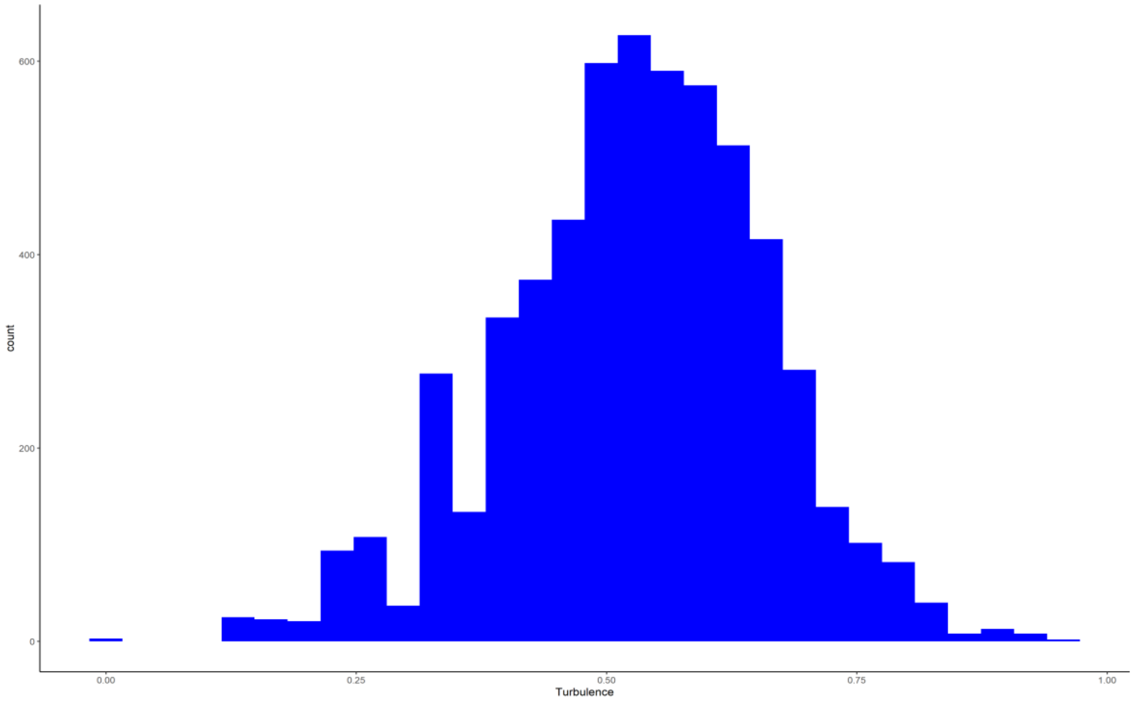

,

**S6 Fig.** Sequence turbulence measures for men

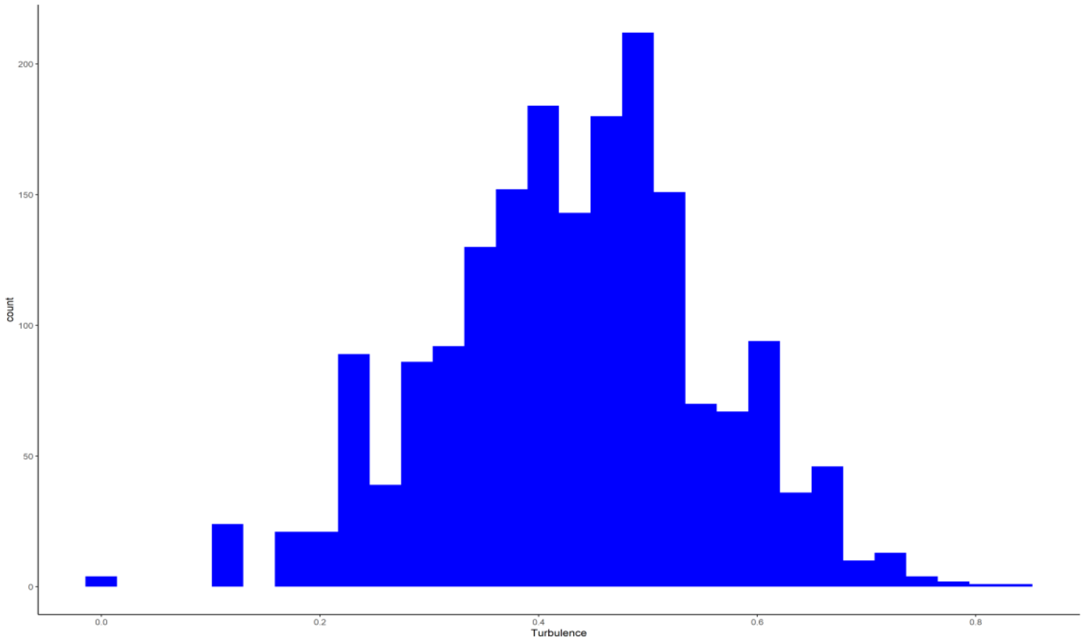

Supplement: Supplementary file 1 — Supplementary Material 1 [file 41598_2026_35960_MOESM1_ESM.pdf]
